# Impact of Minimally Invasive Surgery on Quality of Life and Infertility in Deep Infiltrating Endometriosis

**DOI:** 10.3390/jcm14207256

**Published:** 2025-10-14

**Authors:** Andrei Manu, Elena Poenaru, Florentina Duica, Smaranda Stoleru, Alexandra Irma Gabriela Bausic, Bogdan-Catalin Coroleuca, Ciprian-Andrei Coroleuca, Cristina Iacob, Elvira Brătilă

**Affiliations:** 1 Doctoral School, “Carol Davila” University of Medicine and Pharmacy, 020021 Bucharest, Romania; andrei.manu@drd.umfcd.ro (A.M.); cristina.iacob@drd.umfcd.ro (C.I.); 2 Clinical Hospital of Obstetrics and Gynecology “Prof. Dr. Panait Sârbu”, 060251 Bucharest, Romania; alexandra.bausic@umfcd.ro (A.I.G.B.); ccoroleuca@yahoo.com (B.-C.C.); cip_coroleuca@yahoo.com (C.-A.C.); elvirabarbulea@gmail.com (E.B.); 3 Faculty of Medicine, “Carol Davila” University of Medicine and Pharmacy, 050474 Bucharest, Romania; florentina.duica80@gmail.com

**Keywords:** endometriosis, quality of life, EHP-30, SF-36, EQ-5D, Endometriosis Fertility Index, rASRM, ENZIAN, minimally invasive surgery

## Abstract

**Background**: Endometriosis is a chronic, estrogen-dependent inflammatory disease affecting up to 10% of women of reproductive age. It substantially impacts quality of life (QoL) through pelvic pain, infertility, and psychological distress. Increasing attention has been directed toward patient-reported outcomes and validated QoL instruments, which are essential for understanding the burden of disease and guiding individualized management. **Materials and Methods**: We performed a narrative review of the literature published in the last five years in PubMed, Scopus, Web of Science, and Cochrane Library, focusing on validated QoL instruments, fertility indices, and clinical outcomes after minimally invasive surgery (MIS) for deep infiltrating endometriosis (DIE). **Discussions**: The most widely used QoL instruments are the Endometriosis Health Profile-30 (EHP-30), Short Form-36 (SF-36), and EQ-5D, each providing multidimensional evaluation across physical, psychological, and social domains. Fertility-related prognosis is assessed with the Endometriosis Fertility Index (EFI), while staging of disease severity relies on rASRM and #ENZIAN classifications. Evidence from comparative and cohort studies suggests that both laparoscopic and robotic MIS can improve QoL and reproductive outcomes; however, the magnitude of benefit varies across studies, patient phenotypes, and follow-up periods. **Conclusions**: MIS is an increasingly used therapeutic option for DIE, with growing evidence of improvement in pain and QoL, but current data remain heterogeneous and do not uniformly support superiority over other approaches. Routine incorporation of validated QoL instruments and fertility indices into both clinical practice and research is essential to better stratify patients, support shared decision-making, and optimize long-term outcomes.

## 1. Introduction

Endometriosis is a benign, estrogen-dependent, chronic inflammatory disease affecting up to 10% of women of reproductive age. It is characterized by the presence of endometrial-like tissue outside the uterine cavity [1,2,3]. Despite extensive research into its pathogenesis and heterogeneous clinical manifestations, both diagnosis and management remain challenging [1,2,3].

Women affected by this disease present a very heterogeneous spectrum of symptoms, ranging from simple abdominal and pelvic pain to involvement of other organs such as the abdominal wall, lungs, skin, or, rarely, the central nervous system, with very specific manifestation [1,2,3].

If not diagnosed and treated in time, endometriosis can evolve into an extremely disabling form, leading to the destruction of the affected anatomical structures. Thus, the main effect due to endometriosis is the decrease in the quality of life (QoL) of affected women and the appearance of infertility [4,5,6].

Many recent studies that have reported data on endometriosis have focused on characterizing this disease both from the point of view of symptoms and treatment, as well as from that of possible mechanisms of occurrence and progression. The prevalence of this disease has also been followed over a period of 3 to 5 years, as well as reporting data on the evolution of this disease [7,8,9].

The etiology of endometriosis is not yet fully understood, there are several theories on the occurrence and evolution of the disease, the most accepted being that of retrograde menstruation, where the endometrial-like cells that are able to migrate and implant into various sites under the action of some factors like immune system imbalance, chronic inflammation, localized steroidogenesis and neurogenesis, also genetic, genomic and epigenomic alterations [7,9].

The classification of endometriosis, which is not necessarily done according to the severity of the symptoms, is commonly staged into four categories: minimal, mild, moderate, and severe based on surgical observations (e.g., rASRM) and/or fertility outcomes [6,10,11].

The main symptoms reported by patients suffering from endometriosis are debilitating pain that occurs in various places, such as the pelvis, ovaries (painful ovulation) and the sacral region of the spine, or may occur during processes such as urination, defecation, and during sexual intercourse. Other symptoms are dysmenorrhea, dyspareunia and dyschezia, often associated with a large abdominopelvic mass, as a consequence of a form of deep infiltrating endometriosis (DIE), which is a complex form of endometriosis and can have severe consequences on the quality of life of affected women, but the severity of symptoms is not correlated with the severity of the disease, there are cases in patients with substantial disease that may be asymptomatic. In general, all these symptoms are chronic and often lead to infertility [10,11]. Women with endometriosis may also experience other non-specific symptoms such as headaches, dizziness, fatigue and mood disorders such as anxiety or even depression. All of these symptoms can significantly affect the quality of life of patients [12].

The World Health Organization has recognized endometriosis as a condition with significant social, public health, and economic implications [4]. Although it cannot be prevented, early diagnosis and appropriate management may reduce disease progression and symptom burden. However, diagnosis is frequently delayed—by an average of 6–10 years from symptom onset—because symptoms often overlap with other gynecological and gastrointestinal conditions [13].

Among the most common diagnostic methods, in addition to clinical evaluation of symptoms, are ultrasonography. magnetic resonance imaging and histopathological investigations or, more recently, genomic/genetic analyses. The gold standard is considered laparoscopic visualization of lesions and histopathological confirmation, which also allows surgical excision of affected tissues [14,15].

The classification of endometriosis types has been done according to various systems, depending on the organization or guideline that recommends it. In an article published by Vermeulen et al. (2021) reported that 22 endometriosis classification, staging, and reporting systems were published between 1973 and 2021 [13].

Among the working groups and organizations that have participated in the phenotypic classification of endometriosis, the most recent are the International working group of American Association of Gynecologic Laparoscopists (AAGL), International Society for Gynecologic Endoscopy (ISGE), European Society for Gynaecological Endoscopy (ESGE), European Society of Human Reproduction and Embryology (ESHRE), World Endometriosis Society (WES), along with the European Society of Urogenital Radiology (ESUR), International Society of Ultrasound in Obstetrics and Gynecology (ISUOG) and International Deep Endometriosis Analysis (IDEA) group, which have systematized the classification of this disease into three phenotypes, namely: peritoneal, ovarian (endometrioma) and deep infiltrating endometriosis [14].

The diagnostic approach to endometriosis has evolved significantly, with transvaginal ultrasound (TVUS) now established as a first-line tool for the detection of deep infiltrating endometriosis (DIE). In a comparative study, Baușic et al. demonstrated that TVUS offers diagnostic accuracy comparable to that of magnetic resonance imaging (MRI), particularly in identifying rectovaginal nodules and posterior compartment lesions [15]. Moreover, the application of sonovaginography using ultrasound gel further enhanced the detection of posterior DIE, offering a low-cost, accessible, and reproducible method for mapping lesions preoperatively [16]. These findings support the central role of advanced ultrasound techniques in the initial diagnostic work-up and pre-surgical planning for patients with suspected endometriosis [15,16].

At the molecular level, endometriosis exhibits features that promote local tissue invasion, angiogenesis, and cellular proliferation. A recent molecular study by Coroleucă et al. revealed high expression levels of estrogen receptors (ER), progesterone receptors (PR), the anti-apoptotic marker Bcl-2, and the proliferation marker Ki-67 in ectopic endometrial tissue, suggesting a distinctive molecular signature associated with enhanced survival and implantation capacity [17].

In recent years, surgical management of DIE has increasingly focused on minimally invasive approaches. Robotic-assisted laparoscopy (RALS) combines the benefits of laparoscopy with enhanced dexterity and motion scaling, with broadly comparable short- and long-term outcomes to conventional laparoscopy in selected benign gynecologic indications [18,19,20].

## 2. Materials and Methods

The aim of this review was to evaluate the relationship between medical and surgical interventions in women with endometriosis and their impact on quality of life and reproductive outcomes, by analyzing the most recent publications in this field.

To this end, we conducted a literature search for a narrative review, using keywords such as “endometriosis”, “surgical approaches in patients with endometriosis”, “quality of life in patients with endometriosis”, “therapeutic approaches in endometriosis”, “fertility in patients affected by endometriosis”. Studies reporting original results published in the last 5 years were taken into account; the main specialized search engines used for searches were MEDLINE/PubMed, Google Scholar, Web of Science, Scopus, and Cochrane Library.

We included original research articles written in English that were directly relevant to endometriosis, QoL, and surgical or medical interventions.

We excluded articles without full-text access, articles published in languages other than English, and studies addressing topics outside the scope of this review.

Relevant data according to the inclusion criteria in the study were extracted from the selected articles. The extracted data were synthesized to provide a comprehensive overview of current evidence on QoL and surgical interventions in women with endometriosis.

## 3. Results and Discussion

Following the literature search, the studies that met the inclusion criteria were reviewed and grouped thematically into four main domains: (1) the pathophysiology and clinical manifestations of deep infiltrating endometriosis (DIE); (2) validated tools for assessing quality of life and their application in clinical practice; (3) fertility implications and prognostic indices; and (4) the impact of medical and surgical interventions, with particular attention to minimally invasive approaches. The following subsections summarize and discuss the main findings from the recent literature in each of these domains.

### 3.1. Pathophysiology of Deep Infiltrating Endometriosis

Endometriosis can negatively affect quality of life and lead to infertility, affecting an estimated 190 million women worldwide [21]. Pelvic endometriosis most commonly affects the ovaries, peritoneum, and deep structures such as the bladder or bowel. In deep infiltrating endometriosis, ectopic endometrial cells trigger proliferative activity and a secretory response involving immune and chemical mediators, leading to a chronic inflammatory and fibrotic environment [22]. Despite progress in molecular and imaging studies, the underlying biological mechanisms remain poorly understood [14]. Surgical treatment is often considered the preferred approach when symptomatic lesions infiltrate visceral organs [18,20]. In cases of ovarian endometriomas, exploration, excision of the cavity, and should be performed with excision of the cyst wall and meticulous hemostasis. In cases of deep infiltrating endometriosis affecting the bowel, surgical treatment should consist of excision of endometriosis and the part of bowel involved, plus anastomosis. In cases of deep pelvic endometriosis involving the bladder, surgical resection should be performed after cystoscopy, thereby preserving bladder trigone [23,24]. Laparoscopy has been shown to be safe and allows both accurate diagnosis and complete surgical treatment. It is associated with shorter hospitalization, scarring, and fewer complications compared to laparotomy. Although radical treatment can reduce pain, infertility may persist due to complex pathophysiology, and long-term outcomes remain poorly understood [25]. Fertility is a highly complex biological phenomenon requiring a perfectly synchronized and harmonious interplay between hormones, ova, sperm, and the female reproductive tract. It is also influenced by a variety of psychosocial and environmental factors, and consequently is highly variable between women. It affects 480 million couples of reproductive age and is considered a public health issue. Despite this number, current research has been unsuccessfully attempting to elucidate many aspects of infertility, from initiation mechanisms to risk factors and ethnic disparities, thus preventing access to a more personalized approach to treatment [26,27]. Vast amounts of literature exist regarding infertility treatment, with associated ethics, regulations, and technological advances preventing a complete overview of this topic. Current and future women may be affected by infertility due to initiation of academic life at an increasingly younger age and a worldwide trend towards later age at conception. This trend is exacerbated by a wide range of pathophysiologies affecting the female reproductive tract [28,29]. Minimally invasive video-assisted surgery has gained popularity in the past three decades. The purpose of this surgical technique is to minimize trauma to healthy human tissue and provide a better understanding of anatomy, thus allowing significantly reduced postoperative morbidity [30,31,32].

#### 3.1.1. Burden of Disease

Endometriosis is a complex multiparametric disease that leads to a profound burden on health for women and society. Affecting nearly all aspects of daily living in patients of childbearing age, it is often debilitating for affected individuals and associated with substantial social and economic costs. Endometriosis has been reported in up to 44% of symptomatic women undergoing laparoscopy, with a wide variety of symptoms suggestive of the disease [32]. Among the different types of endometriosis, deep infiltrating endometriosis (DIE) is associated with the greatest impairment of quality of life, comparable in some aspects to gynecologic cancers.

Although some patients remain asymptomatic, DIE may cause chronic pelvic pain, dysmenorrhea, dyspareunia, and infertility [28,32]. Medical therapy to suppress estrogen production is considered the first-line treatment [33,34,35]. If medical treatment is ineffective, contraindicated, or rejected, surgery may be necessary. Recommendations for symptomatic deep endometriosis favour extensive surgery to excise all endometriosis lesions and reduce associated symptoms. Minimally invasive surgery, particularly laparoscopy, has demonstrated feasibility and efficacy in removing DIE nodules, with improvements in pain and quality of life. Treatment options for different phenotypic expressions of endometriosis are summarized in Table 1 [36].

#### 3.1.2. Mechanisms of Infertility in Endometriosis

About 38–50% of patients with deep infiltrating endometriosis (DIE) are affected by infertility and/or subfertility, as revealed by a systematic review [44,45]. The mechanism by which endometriosis occurs as a disease has not yet been fully elucidated. Proposed pathways include peritoneal inflammation leading to ovarian dysfunction, altered folliculogenesis, reduced ovarian reserve, impaired oocyte quality, and tubal obstruction [46]. In addition, ovarian dysfunction can lead to a decrease in ovarian reserve, an inflammatory environment and ovarian endometriosis, which leads to a decrease in oocyte quality or follicle rupture and may be responsible for reduced fertility as demonstrated in cases of mild endometriosis [46].

Endometriosis is well-established as a cause of infertility, but its influence extends into pregnancy, with emerging evidence linking the condition to adverse maternal–fetal outcomes. Frîncu et al. (2021) [47] conducted a comprehensive review comparing pregnant women with and without endometriosis, highlighting elevated risks—most notably preeclampsia, placenta previa, preterm birth, and small for gestational age infants. Their analysis, which included diverse cohort and case–control studies, found odds ratios ranging from 1.2 to 3.1 across these complications, emphasizing the systemic impact of DIE extending beyond conception [47]. These findings underscore that endometriosis-related infertility often necessitates assisted reproductive technologies, which may further compound perinatal risks. This underscores the importance of integrated care: surgical and fertility planning must address not only conception but also optimized prenatal monitoring [47,48].

Reported post-surgical pregnancy and live birth rates range between 50–97%, whereas recurrence of disease or persistent infertility is observed in 10–59% of patients [49,50]. Post-surgery assessment results vary from a rate of pregnancy and/or live births of 50–97%, while recurrent illness or infertility occurs in 10–59% of patients. Hence, the immediate question arises: what is the influence of endometriosis on fertility? Because endometriosis causes inflammation in the peritoneum, the peritoneal environment becomes favorable for endometriosis, while being unfavorable for ovulation and fertilization [51]. Impairment of normal ovulation is attributed to 3 theories: blockage of the ovulatory follicle rupture site at the ovarian cortex, alteration of the follicle size, and alteration of ovarian dysfunction by inflammation in the peritoneum. In addition, endometriosis in some patients shows a decrease in ovarian reserve [52]. However, improper treatment may lead to unnecessary or premature salpingectomy. Resection of the paratubal endometriosis, which adheres to the ampulla of the fallopian tube, has been reported to improve pregnancy rates in those with unexplained infertility [52].

Accurate imaging, particularly TVUS and MRI, remains essential for preoperative planning in infertile women with suspected DIE [49,50,51,52].

### 3.2. Indications for Surgery

Surgical treatment of all visible endometriotic lesions is recommended for patients with deep infiltrating endometriosis, particularly patients with endometriosis-related disorders managed medically with a failure to improve symptoms [53]. Laparoscopic or robotic-assisted surgery is associated with improved quality of life and symptom relief. However, surgery is generally not indicated for patients with stable endometriosis on MRI who are well controlled with medical therapy and have a low to moderate risk of complications. In patients with rectal endometriosis visible on MRI but without bowel symptoms, a trial of medical treatment is also recommended [53,54].

Surgery for endometriosis in patients with bowel symptoms is indicated in cases of chronic pain, obstructive intestinal syndrome, infertility, or documented bowel involvement. Bowel surgery for endometriosis may be performed in specialized centers with extensive laparoscopic expertise, even if they are not tertiary referral centers, while robotic-assisted procedures are usually reserved for high-volume tertiary centers. Optimal management requires adequately trained surgeons to ensure the best outcomes. Similarly, the surgical treatment of urinary tract endometriosis can be undertaken in centers with advanced laparoscopic experience [55,56,57].

#### 3.2.1. Minimally Invasive Surgical Techniques

Several studies have investigated the improvement of reproductive outcomes after surgical treatment for deep infiltrating endometriosis (DIE) affecting the ovaries. Accordingly, it has been suggested that pregnancy and fertility results are still unclear after laparoscopic presacral neurectomy for the treatment of DIE. Future studies concentrated on meta-analyses are required to provide stronger conclusions regarding this surgical technique. Minimally invasive surgery (MIS) represents a surgical technique that involves the use of laparoscopy or robotic surgery to insert miniaturized instruments with a camera. Recently, power morcellators have been introduced to allow the morcellation of larger specimens [58].

Compared with open surgery, MIS is associated with reduced postoperative pain, quicker recovery, earlier return to work and daily activities, and lower postoperative complications. Many surgical procedures in gynecology and surgical treatment modalities for deep endometriosis have been performed with MIS techniques with significant improvement in the patients’ quality of life [59]. Laparoscopic or robotic-assisted power-minimally invasive morcellation of larger endometriosis specimens is now a well-accepted procedure as in other benign pathologies. However, in rare complex cases of malignant nodules, inclusion of a safe large radical open surgical procedure is highly recommended. Surgical treatment modalities of deep infiltrating endometriosis with bowel, bladder, ureter, vagina, and/or pelvic sidewall involvement have also been successfully performed with MIS techniques with excellent surgical outcomes and significant improvement in the patients’ quality of life [60].

MIS for deep infiltrating endometriosis with bladder involvement can maintain functional bladder capacity and reduce the rate of bothersome urinary symptoms 12 months after surgery. Similarly, the surgical treatment of endometriosis affecting the uterosacral ligaments causes improvement of quality of life after 1 year. Accumulative evidence has shown improvement of quality of life in the majority of the patients 1 year after surgery and of dysmenorrhea, dyspareunia, and pelvic pressure symptoms at least 12 months after surgery after laparoscopic or robotic-assisted excision, hysterectomy, or shaving of deep anterior vaginal wall endometriosis [61]. Laparoscopic-colorectal resection is considered a safe and effective procedure in women with rectovaginal endometriosis involving the bowel. Roman et al. [23] conducted a multicenter study including 96 women with rectal DIE, of whom 55 underwent segmental bowel resection and 41 received excision of rectal lesions. Both groups demonstrated significant QoL improvements, although segmental resection was associated with greater relief of global pain, while lesion excision alone was more effective for expulsion pain. Of note, laparoscopic treatment was associated with an earlier return to normal activity than laparotomy [62,63].

#### 3.2.2. Robotic Surgery

Management of deep infiltrating endometriosis (DIE) is multidisciplinary, combining medical, surgical, and psychological approaches, each with specific advantages and limitations. Medical treatment, particularly hormonal therapy, is effective in reducing pain symptoms but has little impact on fertility restoration [34,35]. Fertility is therefore most often attempted after, or in conjunction with, surgical removal of lesions using minimally invasive surgery (MIS). Several prospective and retrospective studies have demonstrated that MIS, whether laparoscopic or robotic-assisted, significantly improves pain outcomes and fertility rates. For example, laparoscopic excision of DIE has been associated with spontaneous pregnancy rates ranging from 45–60% within one year, alongside marked reductions in dysmenorrhea and dyspareunia [54,55,56].

Beyond fertility and symptom control, MIS has a substantial impact on quality of life (QoL). In a prospective cohort study, women with higher baseline QoL scores experienced significantly greater improvements in pain and well-being after surgery, whereas those with severely impaired baseline QoL showed more modest gains, with a subset remaining symptomatic despite complete lesion excision [59]. These findings underscore the predictive value of preoperative QoL assessment and the importance of individualized follow-up. Other cross-sectional studies have reported that a decrease in QoL is strongly associated with worsening chronic pain symptoms, and that improvement in QoL after surgery parallels the degree of postoperative pain relief [64]. Nevertheless, up to 20–30% of women may continue to report impaired QoL even after technically successful MIS, indicating the need for additional supportive or psychological interventions [64].

Robotic-assisted surgery (RAS) has expanded the armamentarium of MIS techniques, particularly for complex DIE requiring extensive dissection or suturing. A retrospective series of 156 patients operated between 2018 and 2021 reported significant postoperative improvements in pain scores and global QoL, as assessed by validated tools including the Endometriosis Health Profile-30 (EHP-30), the EORTC QLQ-C30, and the Numeric Rating Scale (NRS), with benefits maintained at two-year follow-up [65,66]. Importantly, multidisciplinary evaluation and precise preoperative mapping were emphasized as critical for patient selection and surgical planning, as outcomes were optimized when procedures were performed in high-volume, specialized centers [67].

#### 3.2.3. Comparison of Laparoscopic and Robotic Outcomes

Both laparoscopic and robotic-assisted surgery (RAS) are minimally invasive approaches for the management of DIE; while laparoscopy remains the standard technique, RAS provides enhanced dexterity and three-dimensional visualization, which may offer advantages in complex cases [68,69]. The literature search and full-text assessment found eight studies to include in the qualitative synthesis. Four were cohort studies, three were comparative studies reporting on robotic-assisted laparoscopy (RAL) with respect to classical laparoscopy (CL), and one was a meta-analysis. Inclusion/exclusion criteria, comparison outcomes, and sample sizes varied widely between studies. RAS is a safe and feasible surgery for DIE. Even a matched analysis showed that operative time and cycle day count were similar in both groups [70]. The robotic-assisted approach had the additional benefit of a decreased risk in intraoperative and postoperative complications compared with conventional laparoscopy. As for the studies directly comparing robotic with laparoscopic surgery, there was a conflicting tendency in the results concerning the surgeon’s training status. In the beginning, when surgeons were still learning the RAS procedure, longer operative times and a higher blood loss with a robotic approach were reported. Comparatively, when the surgeons were more familiar with robotic techniques, the surgical outcomes between RAS and CL in terms of the operative time, duration of hospitalization, and perioperative complications significantly favored RAS [71].

#### 3.2.4. Operative Time

There are currently no strict recommendations regarding the choice of surgical approach for the treatment of endometriosis, and operative time (OT) remains one of the most debated parameters. Laparoscopy is generally regarded as the first-line minimally invasive (MI) approach, while robotic-assisted laparoscopy has been proposed as an alternative in selected complex cases [72]. A systematic review by Rivero-Moreno et al. [72] pooled available studies and found significant differences in estimated time (ET) and OT that favored the robotic approach; however, heterogeneity in study design and reporting limited the ability to draw firm conclusions when directly comparing laparoscopy and robotics [72,73]. Subgroup analyses indicated that in cases with extensive or deep infiltrating disease, robotic surgery was associated with shorter OT compared with conventional laparoscopy, likely reflecting technical advantages such as improved ergonomics and instrument dexterity [72]. Importantly, surgical experience and center expertise strongly influenced outcomes: centers with established robotic programs reported shorter OT, whereas hospitals without such experience identified lack of referral-center status as an independent risk factor for prolonged procedures [72]. Overall, although MI surgery for DIE tends to be associated with longer OT compared with traditional open approaches, this drawback is offset by a more favorable profile of perioperative and postoperative complications [72,74].

#### 3.2.5. Complication Rates

Reported complication rates after laparoscopic surgery for endometriosis generally range between 0.7% and 4% [74]. However, several studies have noted higher figures, particularly when complex procedures involving bowel, bladder, or urinary tract reconstruction are included, which suggests that earlier estimates may underestimate the true risk in patients with advanced disease [74,75]. By contrast, very low complication rates (<5%) have been consistently observed in endometrioma cohorts, where procedures are less technically demanding and usually performed in isolation [75].

The type of surgical technique and instruments used can also influence complication rates. For example, excessive coagulation with scissor techniques has been linked to thermal damage and subsequent complications, while alternative approaches such as laser dissection have been associated with lower morbidity [75]. Importantly, recurrence rates remain relatively low across most series, and many patients report high satisfaction after surgery despite residual or recurrent complaints, reflecting the significant overall improvement in quality of life (QoL) achieved [74,75]. These findings highlight the need for individualized surgical planning and the potential benefit of adopting advanced energy devices to minimize risks [75].

#### 3.2.6. Recovery Time

Minimally invasive surgical management of deep infiltrating endometriosis (DIE) has been shown to provide sustained improvements in quality of life (QoL) over at least two years of follow-up [76]. However, outcomes are heterogeneous across studies, as the magnitude of QoL improvement depends on the type and extent of surgery performed. For example, a multicenter analysis reported that laparoscopy for bowel- and bladder-infiltrating endometriosis resulted in significantly greater net gains in QoL compared with more limited procedures, reflecting the higher symptom burden addressed in these patients [75,76].

Long-term outcomes have also been reported in a patient-initiated, prospectively maintained registry of women with DIE undergoing surgical treatment. In this registry, patients with persistent pain symptoms prior to surgery experienced substantial and durable improvements in both symptom severity and QoL following operative management, with benefits sustained at two-year follow-up [76]. These findings highlight that although surgical treatment of DIE carries procedural risks, it offers the potential for meaningful and lasting improvements in daily functioning and well-being for appropriately selected patients [76,77].

### 3.3. Validated Scoring Systems

Two validated scores are used in clinical practice to assess the quality of life in women suffering from endometriosis: the EHP-30 and the SF-36. These two questionnaires have practical implications, particularly the EHP-30, which is easy to fulfill, performant and addressed to a specific population. The study of global QoL after minimally invasive surgery is complex given an overall appropriate score to attain the impact of the disease on HRQoL [78]. Most of the previous studies addressed populations without validated scores for assessment of QoL and without validated questionnaires. The study of endometriosis and deep infiltrating endometriosis (DIE) is even more complicated given the large bias of surgery (multidisciplinary or not, robotic or laparoscopic alone, etc.) [78,79]. The Cochrane review analyzing surgical treatment for endometriosis did not find sufficient evidence for minimally invasive surgery on QoL or pain. The efficacy of bowel preparation in the assessment of bowel symptoms was also not found. Endometriosis is a chronic disease impacting the quality of life of women. Besides the health-related quality of life issue, endometriosis is ranked as one of the priority conditions in women’s health research [80,81]. Study on health-related quality of life in women suffering from endometriosis has increased in the past 20 years, but still, there is a lack of well-designed studies on this issue in DIE. Routine assessment of QoL is advised every 3 or 6 months postoperatively, and this QoL assessment could be stored in a database and retrospectively analyzed to address potential improved factors, and at the opposite, unchanged factors after surgery and in the follow-up. Such analyses could be of great help to enhance understanding of endometriosis and its treatment and to improve women’s lives [82,83].

#### 3.3.1. American Society for Reproductive Medicine (ASRM)

The currently used classification of endometriosis is the classification system. The purpose of this classification was to create a universal language to describe endometriosis and serve as a basis for further studies. However, this classification presents some limitations. The major limitation is the inability to consistently predict the presence and degree of pelvic pain based on the findings of the classification. Despite the lack of a consensus definition of the efficacy in the treatment of endometriosis, a consistently reported benefit of surgical or medical approaches is the improvement in health-related quality of life (QoL) [84].

Uterine endometriosis and deep infiltrating endometriosis (DIE) are common conditions in women of reproductive age that may be responsible for infertility and chronic pelvic pain. Women who wish to become pregnant may be treated by surgery to excise endometriosis lesions and restore normal anatomy. The surgical approach can be performed by laparoscopy or laparotomy. Robotic-assisted laparoscopic surgery is an established method of minimally invasive surgery with increased precision. Owing to the complexity of the procedures, the surgical treatment of endometriosis should be performed by specialists experienced in the management of endometriosis. Minimally invasive surgery (MIS) is believed to be associated with a faster recovery and reduced post-operative pain. Moreover, the hemostatic agents used during the surgery are believed to prevent post-operative scarring which could consequently decrease pain and improve the fertility rates. The impact on QoL is the best measure to evaluate the benefit of a treatment for endometriosis [85].

Women suffering from endometriosis report a lower QoL compared to controls. The symptom-based measure Wexner scale correlates well with the health QoL measure Short Form Health Survey (SF36). In a cohort of pre-menopausal women with endometriosis, there are correlations between endometriosis and the emotional and mental fields of the validated questionnaire EQ-5D-3L. The generic SF36 has been used in several studies targeting endometriosis treatment. Nevertheless, disease-validation and translated versions of specific QoL and health resource utilization questionnaires are missing in the literature [86,87]. The QoL measure Endometriosis Health Profile (EHP30) is specific to endometriosis and widely used in Europe. The purpose of EPHEMER is to translate and provide a French adapted version of the EHP30 to allow easy access to this questionnaire in France and French-speaking countries. Moreover, the conception and validation of a QoL and health resource utilization questionnaire composed of validated tools are needed. An objective of the study is to transcribe the existing questionnaires of QoL and health resource utilization with the help of experts [88,89,90].

#### 3.3.2. Enzian Classification

Endometriosis is classified based on its location, with the most frequent being peritoneal surface (Superficial Endometriosis—SE), ovarian endometriosis, and associated with the bowel or bladder, called deep infiltrating endometriosis (DIE) [9]. Endometriosis affects primarily young women of reproductive age (25–35 years), leading to impaired health-related quality of life (HRQoL) due to debilitating symptoms. It is one of the main causes of pain and infertility in women. The most reported symptoms include dysmenorrhea, pelvic pain, dyspareunia, modified gut transit, intestinal pain, infertility, ovarian mass, dysuria, other urinary disorders, and various others. The American Society for Reproductive Medicine (ASRM) score is used for the staging of endometriosis and has been widely adopted. It is a simple method to describe the disease based on the measure and score of each type of location, but it is a subjective scale and has limitations, as it does not correlate well with morphologic affection and prediction of the success of pregnancy. In 2005, a working group in Austria developed the Enzian classification of endometriosis. This classification includes retroperitoneal affection and DIE [86].

Originally, the Enzian classification was complicated, and not very usable. A review of the Enzian classification of 2011 resulted in improved clarity, usability, and understanding of the size categories. The reviewed version of the Enzian classification (2011) consists of retroperitoneal affection (mesosalpinx, round ligament, and broad ligament) and pelvic organs (vesical, rectal, and vaginal endometriosis). The evaluation method of the Enzian classification (2011) includes the description of the location of lesions by compartments (type) and sizes. Studies to evaluate the Enzian classification for peritoneal endometriosis-related symptoms have shown partial correlation with the classification. However, significant links were reported with pain, dysmenorrhea, and deep dyspareunia, indicating that improved Enzian classification (2011) is a good complement to the rASRM classification of endometriosis for the description of DIE lesions [86].

#### 3.3.3. Endometriosis Fertility Index (EFI)

Endometriosis is a complex gynecological disorder affecting 6–10% of women of childbearing age. The considerable individual variation and heterogeneity of this condition make it particularly difficult to study, especially in terms of understanding its origin, evolution, and best treatment options. Dysmenorrhea and infertility were the first symptoms associated with endometriosis, which is currently known as a chronic gynecological disease characterized by the presence of functioning endometrial tissue outside the uterine cavity [90,91]. Endometriosis is associated with infertility in approximately 30–50% of cases. Endometriosis can affect fecundity through several mechanisms, but distortion of the pelvic anatomy appears to be the leading risk factor. In particular, pelvic or peritubal adhesion has been reported to affect the release, collection, and transport of oocytes, thus contributing to infertility [92].

In 2010, a clinical tool for assessing fertility potential in women with endometriosis was proposed. The 10-point EFI score is intended to forecast the postoperative pregnancy rate in women with surgically diagnosed endometriosis who are attempting to conceive without assisted reproductive technology. The EFI incorporates three historical factors and three surgical factors: two estimating residual adnexal function and one estimating the extent of endometriosis. The resultant EFI score has a range of 0 (poor prognosis) to 10 (excellent prognosis), with progressively improved fertility prognosis correlated with a higher final score. Despite the introduction of the Endometriosis Fertility Index two decades ago, EFI has not been widely implemented in clinical practice. In particular, it is not universally applicable to all cases of infertile endometriosis patients, and its rationale is misunderstood, so it may cause risks of surgical overtreatment. A 2020 article suggested that EFI largely retains its utility as a supportive tool in decision for patients with endometriosis [91,93].

### 3.4. Fertility Outcomes

The fertility outcomes of conservative surgery in the treatment of DIE were evaluated. Among the 62 patients who attempted conception via first live offspring pregnancy after excision surgery, 37 (59.7%) achieved pregnancy during the follow-up period. The patients had an average of 13.6 ± 16.2 months of follow-up after surgery. The average age of women at the time of surgery was 34.0 ± 4.7 years. Of the 37 pregnancies achieved, 32 (86.5%) were spontaneous pregnancies, and 5 (13.5%) were assisted reproductive technology pregnancies [94]. Of the 29 patients that delivered after surgery, 18 patients had healthy live births, and one delivered 2 healthy live births (31 deliveries). No pregnancies occurred among the 30 patients without abdominal pain at the time of the last appointment. The pregnancy rates after surgery were also compared between patients with and without the 4 preoperative combined classic factors [95]. Appropriate operation decisions can be made based on each factor. There were no significant differences in pharmacological treatment, length of hospital stays, and major or minor complications between group A and group B postoperatively. Five patients received the drug for 4, 8, and 8 months postoperatively, respectively, once every three months. The patients underwent a mean follow-up of 30.6 ± 20.2 months after surgery. Fourteen patients became pregnant again, including four patients with natural conception for FET and controlled ovarian stimulation and IUI. Among the patients with a follow-up of more than 6 months, the incidence of surgery-successful pregnancy and pregnancy-live birth was 84.0% [94]. The comparison of varied groups showed that the pregnancy/live birth rates were higher in patients of the healthy ovary subgroup than in those of the compromised ovary subgroup after surgery. And the results were consistent in various subgroup analyses based on age and preoperative length of infertility. In addition, there were no differences in the cumulative pregnancy/live birth chance between patients with different syndromes, stages, or operative classifications. Comparing preoperative factors and surgery outcomes between the two groups showed that the body mass index was higher and the preoperative E-2 level was lower in the group of at least 1 healthy ovary. Preoperative anti-Mullerian hormone showed a tendency towards statistical significance. In addition, after receiving OCPs, there was a decrease in CEA-125 and special endometriosis combination results across both groups [96,97].

#### 3.4.1. Spontaneous Fertility Rates

Spontaneous fertility rates in patients with deep infiltrating endometriosis (DIE) remain controversial. Several studies indicate that overall spontaneous fertility rates after surgery for DIE vary from 14% to 68% [9,96,97]. Fertility rates after surgery for DIE also depend on whether additional assistance is used to achieve pregnancy, and the definition of spontaneous fertility varies in different studies. The authors primarily focused on assessing the efficacy of minimally invasive surgery for the treatment of DIE on spontaneous fertility rate in patients without history of previous fertility treatment. This study revealed that surgery for DIE was successful in restoring spontaneous fertility to some infertile patients. In this context, 38.2% of women became spontaneously pregnant after surgery. Many CES patients whose previous surgeries had been performed with traditional surgical methods were recommended to undergo laparoscopic surgery. Although some international scholars have pointed out that laparoscopy is suitable for treating non-ill-controlled deep infiltrating DIE [96]. Considering that even patients with a high degree of DIE can also achieve promising pregnancy outcomes if operated by an expert surgeon, it was hypothesized that this discrepancy could be caused by either different surgical techniques or criteria for choosing patients. It was also hypothesized that patients with a long infertility history, even within the menstrual cycle, were not evaluated for prognosis by a fertility specialist in this case. The design of this study had the goals of gathering updated information on the quality of life and spontaneous pregnancy rates in carefully selected patients 12 ± 2 and 24 ± 2 months after minimally invasive surgery for severe pain and DIE and evaluated the possible influencing factors. In addition, as the major difference between the two groups was chronic pain management, the laparoscopic procedure was consistent with what had been previously described. The above criteria of better quality of life after surgery were observed in patients with severe endometriosis and pelvic pain and were consistent with the results of other studies [95,96,97].

#### 3.4.2. In Vitro Fertilization (IVF) Success Rates

The impact of surgical excision of deep infiltrating endometriosis (DIE) on subsequent fertility and IVF outcomes remains controversial. Zhang et al. (2022) evaluated reproductive and postoperative outcomes in infertile women with DIE and found that surgical excision improved pain and quality-of-life scores, with a postoperative pregnancy rate of 61.8%, including 29.4% conceptions achieved through IVF [96]. Similarly, Bongioanni et al. (2011) observed that ovarian endometriomas may negatively influence IVF outcomes, but appropriate surgical management could improve oocyte retrieval and fertilization rates [97]. Although surgical treatment can be beneficial in selected patients, the indication for surgery before assisted reproduction should be individualized. This consideration is particularly important in young women, where obstetrical factors and tissue integrity may influence reproductive prognosis [98]. In a more recent analysis, Obino et al. (2021) reported that surgery for endometriosis-related infertility may enhance IVF outcomes in certain cases, but overall results remain heterogeneous and not uniformly conclusive [99]. Collectively, current data indicate that surgical management may increase spontaneous conception rates and optimize IVF performance in selected cases, although evidence remains insufficient for universal recommendations.

### 3.5. Improvements in Pain and Quality of Life

Of patients undergoing surgical management for deep infiltrating endometriosis, the proportion who believed that surgery improved their clinical situation was significant and sustained over time, according to research that prospectively studied quality of life evolution in women with deeply infiltrating endometriosis and debilitating pain. Treatment by extensive laparoscopic excision of endometriosis found that minimally invasive surgery for pain in deep infiltrating endometriosis after the failure of medical treatment significantly improved the quality of life and decreased dysmenorrhea and dyspareunia 2 years after surgery. Patients who did not speak out loud did not display a specific profile associated with the improvement score of quality of life domains, and the real benefit of this treatment should be measured depending on the expected improvement before surgery [86].

The results of a study on disability and quality of life in women with endometriosis who underwent surgery are summarized. Work disability and poor quality of life were higher in study participants with endometriosis with only organic complications. Symptoms with a greater unfavorable impact on quality of life were dyspareunia, dysmenorrhea, and depression. Visual analog scale scores for dysmenorrhea before surgery were independently associated with a greater decrease in the quality of life and non-feasibility of surgery. In patients with endometriosis cyst and/or rectovaginal septum, surgery for endometriosis does not improve the disability and quality of life at 2-year follow-up [85].

Laparoscopic sigmoid resection for endometriosis is a safe and efficacious procedure that positively affects quality of life and gastrointestinal symptoms. Quality of life and gastrointestinal symptom scores significantly improve at 1-year follow-up, especially in those with preoperative abnormal scores. Age less than 35 years is associated with a better quality of life before surgery. The quality of life improvement and postoperative dysmenorrhea resolution is not significantly different between groups [90].

#### 3.5.1. Visual Analog Scale (VAS)

A deep infiltrating endometriosis (DIE) is a condition that is often associated with endometriosis, which affects women around the world. This condition can lead to the risk of infertility, reducing the quality of life of the individual. These conditions are predominant in menstruation and affect around 6 to 10% of women 48. Minimal invasive surgery can be done to treat DEE, which in turn can provide benefits to controlling the pain of the patient, improving the quality of life, and improving fertility. The aim of this study was to analyze the effect of the disease on the quality of life of the patient before the surgery and to analyze the effect of the minimally invasive surgery on the quality of life of the individual and infertility techniques 3 and 6 months after the procedure [90].

Embolic disease can be characterized as a leiomyomas or endometrial disorders. Minimal invasive surgery is dedicated to enforcing the elimination of all endometriotic lesions and restoring the anatomical structures changed by endometriosis. This treatment may be done through laparotomy or minimally invasive surgery. Although laparotomy is more common, it is associated with more pain and a longer recovery time. In order to avoid these adverse consequences of laparotomy, minimizing invasive surgery techniques may be more suitable for diagnosis and treatment due to having less blood loss, pain, and complication, and shorter recovery time. In the operative procedure of treatment, endometriomas can be excised, the endometrial nodule conjoined to the rectum can be removed, and the deep infiltration of the uterosacral ligament can be excised as well [100].

This was an observational, longitudinal, and prospective study carried out with women who had a suspected diagnosis of DIE, evaluated by the same specialized physician, in the hospital. A total of 178 patients were indicated for surgical treatment. In the preoperative period, the patient’s age, color, marital status, schooling, professional status, and parity number were assessed. The quality of life was assessed by the Visual Analog Scale (VAS), prior to conducting the surgical procedure 3 and 6 months after the surgical operation [101].

#### 3.5.2. Short Form Health Survey (SF-36)

Endometriosis (EN), a common gynecological disorder, negatively impacts quality of life (QoL) in women. A significant association was demonstrated between the symptoms of endometriosis and QoL assessment in both the physical as well as mental domains, while other sociodemographic characteristics and infertility showed little impact on QoL. Patients were likely to have more symptomatic disease if they had a prior surgery. Moreover, disease recurrence was seen to significantly impact mental component summary of QoL scores [102].

The relationship between the increasing severity of EN, as per the revised American Society of Reproductive Medicine staging criteria, and worsening sub-score of the mental component summary was highlighted. Misinformation regarding the disease showed significant impact on QoL. In the understanding of EN-affected women, there lies a serious unmet need for awareness that may promote timely diagnosis and management of the disorder. Dubious health information, either related to the disease or treatment options as conveyed by lay health promoters, was seen to negatively impact QoL. Emotional distress caused due to clinically compromised disease state of EN, had impact both on physical as well as mental aspect of health QoL [103].

EN, a common gynecological disorder, is characterized by the presence of endometrial-like tissue outside the uterus. Its various manifestations lead to symptomatology that may develop as early as menarche. EN is postulated to have a varied etiology. When symptomatic, EN negatively impacts quality of life (QoL) in women. Apart from morphological changes affecting pelvic anatomy, the inflammatory function of endometriotic lesions leads to a complex milieu inside the peritoneum and eventually infertility in women with the disorder [104].

#### 3.5.3. Endometriosis Health Profile-30 (EHP-30)

Among the validated quality of life questionnaires for patients with endometriosis is the Endometriosis Health Profile-30 (EHP-30). In Brazil, a transcultural adaptation was done from the EHP-30 and it was called EHP-30Br. This was validated, becoming an appropriate questionnaire to be used for evaluating quality of life in patients with endometriosis [86].

The EHP-QoL questionnaire is a self-administered instrument composed of 30 questions, divided into five domains: three specific sub-scales (EHP-Health, EHP-Work, and EHP-Partner) and two non-specific sub-scales (EHP-Coping and EHP-Social). The specific sub-scales refer directly to health (menstrual pain, chronic pelvic pain, dyspareunia, pain in other locations, gastrointestinal symptoms, urinary symptoms, infertility, pain, impact of health on daily life) and work (absence of work, impaired productivity at work); and partner (dyspareunia, change in menstruation, assisted conception, work absenteeism, care for children or domestic chores. Non-specific sub-scales refer to coping strategies (treatments, alternative treatments, avoid life changes) and social (isolation, lack of support); each question refers to a time spent, each one with the extremes 0 to 4. The final score is between 0 (the best quality of life) and 100 (the worst quality of life) [104].

The EHP-30 questionnaire was used to assess the quality of life of women with endometriosis, before and sixty days after endometriosis surgery. Use of the EHP-30 indicated greater improvement in the global domain of quality of life for patients with deep infiltrating endometriosis. In each specific floor, the group analysis by the EHP-30 showed that before and after surgery the quality of life did not differ in the domains of work and partner, and that a difference occurred in the health domains, coping, and social. In the analysis for each form separately, some domains showed difference adherence in the comparative quality of life before and after surgery [105,106].

Endometriosis is a common disease in women. Deep infiltrating endometriosis (DIE) is the most severe form of endometriosis and affects 1 to 5% of women. It is defined by the presence of ectopic endometrial glands and stroma infiltrating over 5 mm, resulting in a chronic inflammatory reaction, forming scar tissue and fibrotic nodules or cysts. Endometriosis is associated with painful symptoms, impaired social and professional quality of life, and fertility issues. It is often diagnosed at a late stage despite its detriment on a woman’s quality of life. Nevertheless, early diagnosis and treatment means could limit impact and irreversible consequences on quality of life and the fertility of patients. Medical treatment can relieve the symptoms of endometriosis but only temporarily as, upon stopping the therapy, the pain recurs. Surgery allows the complete excision of the disease while considering the preservation of patients’ organs and fertility. Minimally invasive surgery (MIS) is now the gold standard for the management of the disease. There is however no data regarding objective and subjective postoperative Video Quality of Life scores in patients suffering from pain and DIE treated by MIS, including information on the nature of DIE nodules. Pain and quality of life evaluation scales should be used as routinely preoperatively and postoperatively to assess the results and prevent a delay in treatment for other patients.

This retrospective observational study was conducted in a single tertiary university centre, examining the long-term consequences of minimally invasive surgical (MIS) treatment regarding quality of life (QoL) and fertility in patients suffering from deep infiltrating endometriosis (DIE) and pain. This study included all patients suffering from pain and DIE between 2013 and 2016 after failed medical treatment. Medical conditions were collected (general and obstetrical). Quality of Life index was assessed using the Visual analogue scale raised pain day/month, END-QoL, EQ-VAS, EQ-5D-5L, OVI-R, and BDI-II. The results of this study presented a significant statistical improvement in all QoL indexes and indices, with a mean decrease of −8.82 (42.10%) for VAS, 52.15 points for END-QoL score (40.74% to 12.60%), 0.84 points for EQ-VAS (43.85% to 0.43 points), and 0.09 points for EQ-5D-5L weaning effect (45.34% to 0.02 points) concerning DIE symptoms. The number of patients with very bad and bad OVI-R and BDI scores decreased statistically significantly from preoperative to postoperative. The type of surgery was not associated with a difference in any postoperative indexes of QoL [104,105,106,107].

## 4. Conclusions

Deep infiltrating endometriosis (DIE) remains one of the most challenging benign gynecological diseases, with a profound impact on women’s quality of life, reproductive potential, and overall well-being. Advances in imaging and classification systems have improved diagnostic accuracy and surgical planning, yet therapeutic decisions continue to require an individualized and multidisciplinary approach. Medical therapy, particularly hormonal agents, provides effective symptom control for many women, but its limitations in fertility restoration and long-term disease modification highlight the continued need for surgical options.

Minimally invasive surgery (MIS), including both laparoscopic and robotic-assisted approaches, has emerged as the preferred surgical strategy in symptomatic patients refractory to medical treatment. Evidence consistently shows that MIS improves pain, reduces dysmenorrhea and dyspareunia, and enhances quality of life, with benefits that are sustained over several years of follow-up. Furthermore, fertility outcomes following lesion excision are favorable, although heterogeneous, and remain influenced by multiple clinical and patient-related factors. Comparative data suggest that laparoscopic and robotic-assisted surgery achieve broadly similar outcomes in terms of symptom relief and QoL improvement, with robotic platforms potentially offering technical advantages in selected complex cases. Significantly, while MIS is generally associated with longer operative times than open surgery, this is offset by lower complication rates, shorter hospitalization, and faster recovery.

Nevertheless, outcomes are not uniform across all patient groups. A subset of women continue to experience persistent symptoms or impaired quality of life despite technically successful surgery, emphasizing the multifactorial nature of endometriosis and the importance of ongoing medical, psychological, and fertility support. Current evidence remains limited by variability in study design, small sample sizes, and differences in outcome reporting. High-quality, prospective, and comparative studies are still needed to clarify the relative benefits of conservative versus radical excision, as well as laparoscopic versus robotic techniques, and to establish standardized criteria for patient selection. Ultimately, optimal care for women with DIE will depend on individualized treatment strategies, guided by multidisciplinary expertise, validated outcome measures, and the integration of surgical and non-surgical modalities.

## Figures and Tables

**Table 1:** The endometriosis treatment proposed for various phenotypic expression of disease.

Phenotypic Expression of the Disease	Proposed Treatment	References
Superficial Peritoneal Endometriosis (SPE): Phenotype: Minimal/mild disease with surface implants on the peritoneum.	**Medical therapy:** - Combined oral contraceptives (COCs) - Progestins (e.g., norethindrone acetate, dienogest) - GnRH agonists or antagonists (add-back therapy may be required) - LNG-IUS (Levonorgestrel intrauterine system)	[4,5]
	**Surgical therapy:** - Laparoscopic ablation or excision may be considered if medical therapy fails or if diagnosis is uncertain	[36]
Ovarian Endometriomas Phenotype: Cystic ovarian lesions containing endometrial-like tissue (chocolate cysts).	**Medical therapy:** - May reduce cyst size and pain but usually not curative.	[34,37]
	**Surgical therapy:** - Laparoscopic cystectomy (preferred over drainage or coagulation) - Avoid repeated surgeries due to risk of diminished ovarian reserve.	
Deep Infiltrating Endometriosis (DIE): Phenotype: Lesions penetrate > 5 mm under the peritoneal surface, often affecting uterosacral ligaments, bowel, bladder, ureters.	**Medical therapy:** - Non-steroidal anti-inflammatory drugs used for pain management. - Limited efficacy for severe structural disease or organ involvement.	[38,39,40]
	**Surgical therapy:** - Complex, multidisciplinary laparoscopic excision may be needed.	
Adenomyosis (related phenotype): Phenotype: Endometrial tissue within the myometrium; often co-exists with endometriosis.	**Medical:** - Progestins - GnRH agonists - LNG-IUS - COCs	
	**Surgical:** - Conservative surgery (for focal adenomyomas) - Hysterectomy (definitive treatment for women not desiring future fertility)	[41,42,43]

## Data Availability

The data are available from the corresponding author upon reasonable request.

## Abbreviations

The following abbreviations are used in this manuscript:

QoL	Quality of Life
DIE	Deep Infiltrating Endometriosis
SF-36	Short Form-36
EFI	Endometriosis Fertility Index
rASRM	revised American Society for Reproductive Medicine
AAGL	American Association of Gynecologic Laparoscopists
ISGE	International Society for Gynecologic Endoscopy
ESGE	European Society for Gynaecological Endoscopy
ESHRE	European Society of Human Reproduction and Embryology
WES	World Endometriosis Society
ESUR	European Society of Urogenital Radiology
ISUOG	International Society of Ultrasound in Obstetrics and Gynecology
IDEA	International Deep Endometriosis Analysis
RAL	Robotic-Assisted Laparoscopy
TVUS	Transvaginal Ultrasound
MRI	Magnetic Resonance Imaging
ER	Estrogen Receptors
COCs	Combined Oral Contraceptives
LNG-IUS	Levonorgestrel Intrauterine System
RAS	Robotic-Assisted Surgery
CL	Classical Laparoscopy
EHP-30	Endometriosis Health Profile-30
EORTC	European Organization for Research and Treatment of Cancer
NRS	Numeric Rating Scale
OT	Operative Time
ET	Estimated Time
MIS	Minimally Invasive Surgery
HRQoL	Health-Related Quality of Life
ASRM	American Society for Reproductive Medicine
IVF	In Vitro Fertilization
VAS	Visual Analog Scale
EN	Endometriosis
